# Mixing Energy Drinks with Alcohol is Related to Alcohol Problems Over Time Among Reserve and National Guard Soldiers

**DOI:** 10.1093/milmed/usaf632

**Published:** 2026-01-20

**Authors:** Bonnie M Vest, Mala McCormick-Cisse, D Lynn Homish, Gregory G Homish

**Affiliations:** Department of Family Medicine, State University of New York at Buffalo, Buffalo, NY 14203, United States; Department of Community Health and Health Behavior, State University of New York at Buffalo, Buffalo, NY 14214, United States; Department of Community Health and Health Behavior, State University of New York at Buffalo, Buffalo, NY 14214, United States; Department of Community Health and Health Behavior, State University of New York at Buffalo, Buffalo, NY 14214, United States

## Abstract

**Introduction:**

The potential consequences of alcohol mixed with energy drinks (AMED) consumption are of concern, but research to date has yielded conflicting results on how this mixing relates to alcohol outcomes. How AMED might relate to particular at-risk populations, such as military personnel, and the longitudinal consequences are unknown. This research examines the relationship between AMED use with alcohol problems using 6 years of data, among Reserve and National Guard (R/NG) soldiers, a population at risk for both heavy use of caffeine and alcohol.

**Materials and Methods:**

The current analyses used 6 years of annual survey data, collected from soldiers (*n* = 485) participating in Operation: SAFETY, a longitudinal cohort study of U.S. Army R/NG soldiers and their spouses. Generalized estimating equation models were used to examine the relationship between past-year use of energy drinks mixed with alcohol (yes/no) on the likelihood of having an AUDIT score ≥8 (yes/no), indicative of problems with alcohol. Adjusted models controlled for age, sex, and symptoms of PTSD. The study was approved by the Institutional Review Board at the University at Buffalo, and vetted by the Army Human Research Protections Office, the Office of the Chief, the Army Reserve, and the Adjutant General of the National Guard. All participants provided informed consent to participate.

**Results:**

At the first assessment, over half of the sample reported AMED in their lifetime, and 14.4% reported AMED in the past year. Reporting past-year mixing of energy drinks with alcohol was significantly related to higher odds of likely problems with alcohol (Odds Ratio (OR), 2.01; 95% CI, 1.51-2.66; *P* < .001) over time (**Table 1**). This remained true in adjusted models, after accounting for sex, age, and PTSD symptom levels (Adjusted Odds Ratio (AOR), 1.88; 95% Confidence Interval (CI), 1.39-2.54; *P* < .001).

**Conclusions:**

Soldiers are already at greater risk for problems with alcohol compared to their civilian counterparts; the current analyses suggest that using energy drinks combined with alcohol may increase this risk for some individuals. Further research is needed to explore the context in which energy drinks and alcohol are used to inform future prevention messaging and interventions.

## INTRODUCTION

Problematic alcohol use and its related health outcomes are well-established.^1^^,^^2^ However, the potential consequences of consuming alcohol mixed with energy drinks (AMED) remain understudied and are of growing concern. Previous findings on AMED consumption are inconsistent.^3^ Some studies suggest that, compared to alcohol only consumers, individuals who consume AMED engaged in more drinking days in the past month, consumed more alcohol during typical and heavy drinking (studies use varying terminology to describe alcohol use, but generally define heavy episodic and/or binge drinking as past month consumption of 5 or more drinks (males); 4 or more drinks (females) in the same drinking occasion^4–6^^,^^12^^,^^14^; heavy drinking as binge drinking at least 2 days per week^14^; and hazardous alcohol use as having an AUDIT score greater than or equal to 8^15^) episodes, and consumed alcohol for longer durations during these episodes.^4–6^ However, within-subject comparisons of AMED and alcohol-only occasions reveal no difference in alcohol consumption or perceived intoxication and even indicate decreased alcohol consumption during the AMED occasions.^5^ Much of this current research is cross-sectional and primarily limited to adolescents and young adults in college settings.^6–10^ It is not known, therefore, what the longitudinal consequences of AMED might be, and how findings might relate to other at-risk populations, such as ­military personnel.

Examining AMED among military personnel is important due to a high prevalence of problematic alcohol use and overuse of caffeine among service members. A nationally representative study of veterans indicated a lifetime prevalence of alcohol use disorder (AUD) of 41%, with past-year AUD affecting 10.5%.^11^ Among those on active duty in the Army, recent analyses of the Health-Related Behaviors Survey found prevalence rates of heavy episodic drinking and possible alcohol use disorder of 28.2% and 33.1%, respectively.^12^ Reserve and National Guard soldiers may be at similar or greater risk for problems with alcohol; authors of a longitudinal analysis studying a cohort of Reserve and National Guard (R/NG) soldiers reported that among a full pre-deployment sample, 12.6% had a positive alcohol dependence screening.^13^ Past month binge and heavy drinking are also prevalent among Reserve and National Guard soldiers with rates of 29.0% and 7.4%, respectively.^14^ ­Hazardous alcohol use among military populations is concerning and is associated with negative social, physical, and psychological health outcomes.^15^

The heavy consumption of caffeine, especially in the form of energy drinks, among military personnel is also concerning. According to Toblin et al. 75.7% of soldiers in their sample reported past-month energy drink consumption, with 16.1% reporting high consumption rates (at least 2 drinks daily or more than 24 oz/day).^16^

Data from the 2011 Health-Related Behaviors Survey of Active Duty Military Personnel show that 21% reported consuming AMED in the past month.^17^ However, AMED use among military samples differs based on age, biological sex, and military position,^18^ with younger, male, infantry, and airborne soldiers at the greatest risk.^19^ Given the high prevalence of energy drink use and the well-documented research on problematic alcohol use within military populations, a longitudinal analysis of AMED use and its associated outcomes is warranted. The objective of this research is to examine the longitudinal relationship between mixing energy drinks and alcohol with alcohol problems using 6 years of data, among Reserve and National Guard soldiers, a population at risk for both heavy use of caffeine and alcohol.

## METHODS

### Participants and Procedure

Data were drawn from Operation: SAFETY (Soldiers And Families Excelling Through the Years), a longitudinal survey-based study that examined the health and well-being of USAR/NG soldiers and their partners (*N* = 418 couples). Methods for data collection and recruitment have been published in detail elsewhere.^20–22^ Participants were initially recruited in 2014-2015 at in-person events across 47 U.S. Army Reserve and National Guard (USAR/NG) units in New York State. Study team members were invited to drills and trainings at units across the state and introduced the study, and screened participants for eligibility. Once participants enrolled in the study, they were surveyed on an annual basis thereafter over 6 years. While both spouses had to agree to participate initially and at least 1 spouse had to be currently serving in the USAR/NG, participants were retained in the study over time regardless of whether they left the military or were no longer with their original partner. Surveys were electronic via a secure HIPAA-compliant software, and each partner had separate passwords and log-in information. Surveys could be completed either in person at the university or at a time and location of the participant’s choosing. Both partners were compensated for each survey completion (T1: $60 each; T2: $70 each; T3: $70 each; T4: $80 each; T5: $90 each; T6: $90 each) for a total of $460 per individual/$920 per couple over the full study. The study was approved by the Institutional Review Board at the University at Buffalo, and vetted by the Army Human Research Protections Office, the Office of the Chief, the Army Reserve, and the Adjutant General of the National Guard. All participants provided informed consent to participate and were compensated for completion of each survey. Retention in the study over the 6 years was excellent (only 24 couples did not complete the 6th assessment (5.7%)). Notably, only 5 couples (1.2%) have not completed any of the 5 follow-up assessments. Participants were engaged over the study period through mailed newsletters, birthday and holiday cards, and attentive personalized follow-up from research staff.

The current analyses use 6 years of annual survey data from all male and female soldier participants (*N* = 485). Soldiers were primarily male (79.8%; *n* = 387) and were 31.5 (SD = 6.5) years of age on average at baseline. Soldiers self-reported their race as follows: 80% (*n* = 388) White non-Hispanic, 5.2% (*n* = 25) Black non-Hispanic, 8.7% (*n* = 42) Hispanic, and 4.5% (*n* = 22) other and/or multiple race. Participants reported an average of 9.2 (SD = 6.0) years of military service at baseline.

### Measures

The current analyses used the following measures:

#### Alcohol mixed with energy drinks

Our primary predictor was the past-year mixing of alcohol with energy drinks. At timepoints 2 and 3 participants were asked “Have you mixed alcohol with an energy drink, energy shot, or energy mixer?”, measured on an 8-point scale from “0 Never, 1 Tried before but not in the last year, 2 A few times per year, 3 Once per month, 4 Several times per month, 5 At least weekly, 6 A few times per week, 7 Daily.” Responses were dichotomized into Yes (rating 2-6) or No (rating 0-1) for past year use. At timepoints 4-6, the question was revised to ask 3 separate questions about frequency of mixing (1) energy drinks, (2) energy shots, or (3) energy mixers with alcohol. These responses were again dichotomized into a single yes/no item for the past year AMED, based on a positive response to mixing in the past year for any form of energy drink, shot, and/or mixer. The AMED variable was dichotomized for 2 reasons: the differential measurement across timepoints, and the low frequencies of individuals endorsing some of the frequency ratings, which meant we did not have sufficient power to examine these groups separately.

#### Alcohol problems

Our primary outcome was a dichotomous indicator of likely problems with alcohol. Alcohol problems were assessed at each timepoint using the Alcohol Use Disorders Identification Test (AUDIT), which consists of 10 items that relate to potential problems with alcohol in the past year. Scores range from 0-40 (α = 0.78) and scores ≥8 indicate likely alcohol problems.^23^^,^^24^ The dichotomous outcome compared those with a score <8 to those with a score ≥8, as that cutpoint is indicative of clinically significant potential problems with alcohol.

#### PTSD symptoms

Due to strong relationships between PTSD symptoms and alcohol problems among military members,^25^ models controlled for PTSD. Symptoms of PTSD were assessed at each timepoint with the PTSD Checklist,^26^ adapted to map to DSM-5.^27^ The checklist consists of 20 items that ask participants about past-month PTSD symptoms, rated from 0 (*not at all*) to 4 (*extremely*), with an overall range of 0-80 (α = 0.95). Greater scores indicate greater symptoms of PTSD.

#### Demographics

Additionally, models controlled for participants' self-reported sex (male/female) and age.

### Data Analysis

The longitudinal relationship between past-year AMED and alcohol problems (i.e., AUDIT score ≥8) was examined using generalized estimating equations (GEE) models. The GEE model estimates represent the aggregate of within-timepoint relations across all waves, which maximizes the use of available data from each timepoint and accounts for correlation among participants' responses over repeated measures.^28–30^ For these models, we used a logit link function (due to the dichotomous nature of the outcome variable), and a binomial family function and exchangeable correlation structure. Adjusted models controlled for sex (constant), age (time-varying), and PTSD symptoms (time-varying).

## RESULTS

At time 2 (the first time AMED was assessed), over half of the sample reported AMED in their lifetime. Specifically, 37.1% (*n* = 147) reported having tried mixing but not in the past year, 11.6% (*n* = 46) reported mixing a few times a year, and 48.5% (*n* = 192) of participants reported never having mixed alcohol and energy drinks. Frequencies of once per month or more were endorsed by 1% or fewer of participants. This pattern generally continued across each wave of data. At times 4, 5 and 6, the AMED questions were broken out to separately ask about mixing of energy drinks, shots, and mixers with alcohol. At all time points, mixing of energy drinks (e.g., Red Bull, Monster, Amp) with alcohol was the most commonly endorsed form of mixing; 1% or fewer reported past-year mixing of energy shots or ­mixers with alcohol at all time points.

Our primary predictor, the overall prevalence of reported past-year mixing of energy drinks with alcohol, generally decreased in the sample over time from 14.4% at time 2 to 8.5% at time 6 (Odds Ratio (OR), 0.85; 95% Confidence Interval (CI), 0.77-0.94; *P* < .01).

PTSD symptom scores at baseline averaged 9.7 (SD = 12.1); the average score at time 6 was 11.8 (SD = 16.0) (OR, 1.01; 95% CI, 1.01-1.01; *P* < .001). The prevalence of likely problems with alcohol (AUDIT score ≥8) ranged from 14.8% to 18.4% across timepoints, but was not significantly different over time (OR, 1.05; 95% CI, 0.99-1.10).

Reporting past-year mixing of energy drinks with alcohol was significantly related to higher odds of likely problems with alcohol (OR, 2.01; 95% CI, 1.51-2.66; *P* < .001) over time (**Table 1**). This remained true in adjusted models, after accounting for sex, age, and PTSD symptom levels (Adjusted Odds Ratio (AOR), 1.88; 95% CI, 1.39-2.54; *P* < .001).

**Table 1.** Unadjusted and Adjusted Results of Generalized Estimating Equations examining Energy Drinks and Alcohol Mixing on Alcohol Problems Over 6 Years (AUDIT score ≥8)

Variable	OR (95% CI)	AOR (95% CI)
Past year energy drink-alcohol mixing	2.01*** (1.51-2.66)	1.88*** (1.39-2.54)
Sex	—-	0.50* (0.28-0.88)
Age	——	0.97*** (0.94-1.00)
PTSD symptoms	——	1.03*** (1.02-1.04)
Time	1.06 (0.99-1.14)	1.09 (0.99-1.18)

Past year energy drink-alcohol mixing: 0 = no; 1= yes. Sex: 0 = male; 1 = female.

* *P* < .05.

** *P* < .01.

*** *P* < .001.

## DISCUSSION

Among a sample of male and female USAR/NG soldiers, AMED consumption was prevalent, but decreased over time. However, past-year AMED use was associated with increased odds of clinically significant alcohol problems over 6 years. This association remained significant after accounting for soldiers' sex, age, and PTSD symptom levels. AMED consumption among our sample was most commonly reported as happening only a few times per year; these findings suggest that even infrequent AMED consumption may contribute to increased odds of having clinically significant alcohol problems for some individuals.

Soldiers are already at an increased risk for problematic alcohol use compared to their civilian counterparts.^15^ Furthermore, while energy drinks are often perceived as beneficial for enhancing mental alertness and physical endurance among military populations, our findings are consistent with previous research indicating that energy drink use may have adverse effects on alcohol use behaviors and overall health.^16^^,^^18^^,^^19^ This study extends prior research among other high-risk civilian and military populations, and to our knowledge, is among the first to demonstrate a relationship between AMED use and more distal alcohol use outcomes.

There are several limitations to consider when interpreting the present findings. First, participants were recruited from units within New York State and were either married or living with a partner as if married at baseline. While this may limit the generalizability of our findings, half of all reservists are married, and our sample’s baseline demographics closely align with national data on USAR/NG soldiers.^31^ Second, all the data were self-reported, introducing the potential for response bias. However, a confidential survey with validated measures was used to mitigate this concern. Finally, questions about AMED were not identical across timepoints, leading to a need to collapse responses into yes/no for past year use. Future studies might consider examining how energy drink use generally relates to AMED likelihood and frequency. Additional work is also needed using more nuanced measures of AMED, including frequency, and how it relates to problems with alcohol.

Due to the increased risk of alcohol use-related problems among this population, the use of AMED among USAR/NG soldiers is of concern and warrants further evaluation. Next steps should include exploring the context in which AMED consumption occurs among military personnel. While our findings suggest an association between long-term AMED and increased odds of clinically significant alcohol problems for some individuals, even when AMED occurs infrequently, additional research is needed to clarify this relationship and to inform future prevention messaging and interventions.

## Data Availability

The data underlying this article cannot be shared publicly due to the need to protect the privacy of individuals who participated in the study. The data will be shared on a reasonable request to the corresponding author.

## INSTITUTIONAL CLEARANCE

Does not apply.
